# Laser-Assisted Robotic Roller Forming of Ultrahigh-Strength Steel QP1180 with High Precision

**DOI:** 10.3390/ma16031026

**Published:** 2023-01-23

**Authors:** Junying Min, Jincheng Wang, Junhe Lian, Yi Liu, Zeran Hou

**Affiliations:** 1 School of Mechanical Engineering, Tongji University, Shanghai 201804, China; 2 Shanghai Key Laboratory for A&D of Metallic Functional Material, Tongji University, Shanghai 200092, China; 3 Department of Mechanical Engineering, Aalto University, 02150 Espoo, Finland

**Keywords:** laser-assisted forming, ultrahigh-strength steels, sharp bending radius, springback, thermo-mechanical model

## Abstract

Laser-assisted forming provides a perfect solution that overcomes the formability of low-ductility materials. In this study, laser-assisted robotic roller forming (LRRF) was applied to bend ultrahigh-strength steel sheet (a quenching and partitioning steel with a strength grade of 1180 MPa), and the effects of laser power density on the bending forces, springback, and bending radius of the final parts were investigated. The results show that LRRF is capable of reducing bending forces by 43%, and a compact profile with high precision (i.e., a springback angle smaller than 1° and a radius-to-thickness ratio of ~1.2) was finally achieved at a laser power density of 10 J/mm^2^. A higher forming temperature, at which a significant decrease in strength is observed, is responsible for the decrease of forming forces with a laser power density of higher than 7.5 J/mm^2^; another reason could be the heating-to-austenitization temperature and subsequent forming at a temperature above martensitic-transformation temperature. Forming takes place at a higher temperature with lower stresses, and unloading occurs at a relatively lower temperature with the recovery of Young’s modulus; both facilitate the reduction of springback angles. In addition, the sharp bending radius is considered to be attributed to localized deformation and large plastic strains at the heating area.

## 1. Introduction

Considering safety and lightweighting, ultrahigh-strength steels (UHSS) are frequently used in thin-walled structures. The battery-pack housing is an important application of UHSS in electric vehicles (EV). The battery-pack housing not only demands safety and lightweighting to protect the battery from being damaged by external collisions, but also requires a compact design to increase the packing space in battery packs. However, when UHSS is formed to thin-walled and compact structures with small bending radius, it can be a bit challenging. The inner bending radius to sheet thickness (radius-to-thickness, *R/t*) ratio is an important criterion to evaluate the bendability of materials. A smaller *R/t* ratio in thin-walled structures typically implies larger energy-absorption rate and, therefore, better anti-intrusion abilities [1]. In contrast to low-carbon steels, UHSS are characterized by 3–6 times higher yield strength, 3–5 times lower elongation, and thus 3–4 times larger *R/t* ratio [2]. Some one-step forming processes, for example, stamping and press-brake bending, lead to the inevitable formation of defects, such as significant springback [3] and even cracks [4]. Although multi-pass forming strategies like incremental forming and roller forming are feasible alternatives to avoid these shape defects to some extent through the cumulative plastic-deformation mechanism [5], the formation method of ultrahigh-strength components with small bending radii is still limited [6].

Forming at elevated temperatures is another feasible approach to ameliorate the formability of UHSS, whereas the conventional hot stamping processes by continuous blanking, heating, stamping, and quenching are always faced with the lack of flexibility for small-batch production. Consequently, innovations in the heating approach of heat-assisted forming processes, more specifically, the applications of some particular energy sources in forming processes [7,8], are quite prevalent in the recent two decades. For example, Li et al. [9] developed an induction heating-assisted incremental-forming process and found that forming at a temperature of 700 °C improved the geometric accuracy of Ti-6Al-4V alloy sheets significantly due to better ductility. Xiao et al. [10] proposed an electrically assisted electromagnetic-forming process and improved the forming limit of 7075 aluminum alloy by nearly 40% compared to the conventional electromagnetic-forming method. As a high efficiency and flexibility heating source, the laser has gained more attention in the field of forming, as well as in many other fields [11]. Laser-assisted forming processes possess a superior ability to overcome the bottleneck of low-formability materials, reducing the load capacity of forming facilities, as well as realizing property-controlled forming. Mohamadi et al. [12] reported that laser-assisted single-point increment-forming processes not only improved the formability of AA2024-T3 sheet, but also ensured the geometrical accuracy due to partial heating and deformation, as well as the real-time stress release during forming. Liverani et al. [13] introduced laser heating prior to bending process to reduce the hardness and enhance the ductility of the material at the bending area, which not only eliminated the crack defects in traditional cold-bending processes, but also modified the local microstructure of the final product. Gisario et al. [14] performed investigations on the process parameters of laser-assisted bending and concluded that this process was capable of forming titanium and aluminum alloys with a large bending angle and a sharp bending radius. Kant et al. [15] proved that the combination of laser-bending and external mechanical load affected the stress-strain distribution significantly, with little effect on the temperature distribution, and the factors relating to the springback angle in laser-assisted bending, such as laser scanning speed, laser spot dimension, and laser power, were further studied. Guo et al. [16] built an analytical model to predict the bending angle of a pressure-assisted laser-forming process considering both the thermal stress and mechanical stress. Guo et al. [17] designed a laser-assisted four-point bending platform by which a high-stiffness structure with large curvature, smaller springback angle, and better forming quality was obtained at smaller bending forces. To predict the springback angle of laser-assisted bending, which was influenced by multiple process parameters, e.g., laser power, scanning speed, and scanning passes, Ponticelli et al. [18] established a fuzzy logic-based model to describe the inherent uncertainties in laser-assisted forming and to further assess the optimal process parameters. Despite many investigations on the process parameters of laser-assisted forming processes, there is still an insufficient understanding of the mechanical behavior accounting for the final-part profiles in forming with synchronized laser heating.

In this study, a laser-assisted robotic roller forming process (LRRF) was applied to bend an ultrahigh-strength steel (QP1180). The effect of laser power density on the bending profiles of final parts were investigated by experiment, and the reduction of bending forces and springback, as well as the formation of a small bending radius, were further explained using thermo-mechanical modeling.

## 2. Materials and Methods

### 2.1. Laser-Assisted Robotic Roller Forming Experiments

**Figure 1:**
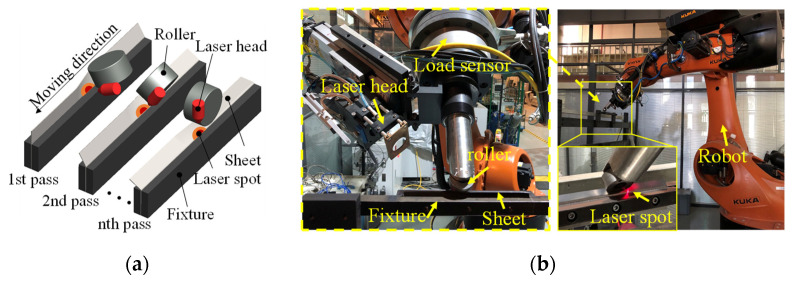
Laser-assisted robotic roller forming, (**a**) schematic of the forming process, (**b**) experimental setup.

**Figure 2:**
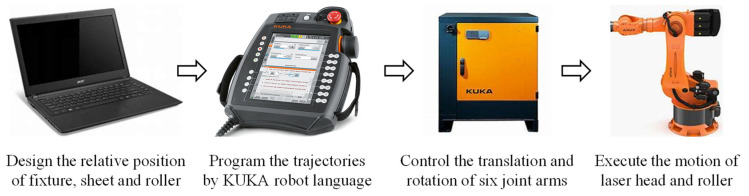
Experimental procedure of LRRF process.

QP1180 steels prepared by a two-step quenching and partitioning process by BaoSteel were investigated in this study. The as-received, 1-mm-thick metal sheets consisted predominantly of martensite, ferrite, and retained austenite [19]. QP1180 steel sheets were cut into 250 mm × 60 mm strips by laser cutting; afterwards, the strips were clamped onto the fixture and ready for the subsequent forming procedure. An LRRF process was applied to bend the strips into nearly 90-degree L-shaped parts by multiple forming passes [4,20]. The schematic of LRRF and the corresponding experimental facilities can be found in Figure 1. In the LRRF process, a high-power laser source is imposed 25 mm in front of the roller to preheat the strips to an elevated temperature, followed by the mechanical bending via the direct contact between the roller and the strips. The laser head and the forming roller are driven by one six-axis heavy-duty industrial robot (KR600, KUKA, Augsburg, Germany) to realize synchronous motion. The trajectories of the roller are firstly designed according to the relative position of the fixture, sheet, and roller, and then they are embedded into the KUKA smartPAD teach pendant using the KUKA robot language (KRL). The programmed trajectories are defined by the translation and rotation of a local Cartesian coordinate system, which is introduced at the end of the roller, relative to the global Cartesian coordinate system. The system controller is then capable of manipulating the motion of joint arms with six degrees of freedom (DOF). The flow chart of the process is shown in Figure 2.

There are large amounts of process parameters affecting the forming process and profiles of the final part, such as laser power, laser spot size, laser scanning speed, forming passes, and angle increment; among them, temperature is generally one of the most important factors. Laser heating induces significant temperature gradients, and it is also difficult or impossible to measure the overall temperature field. Therefore, the direct description on the relationship between temperature and part profiles seems to be infeasible. However, temperature is highly dependent on laser power density, which can be calculated according to the laser power, laser spot size, and laser scanning speed. Therefore, this study focuses on the effect of laser power density on the mechanical behavior during LRRF and the final part profiles. The dimension of the laser spot was 4 mm × 2 mm to ensure adequate heating at the bending corner. A constant moving speed combining various laser powers were adopted in this study to obtain a range of different laser power densities. Specifically, the laser scanning speed, which is also the moving speed of the roller, was 30 mm/s, and the laser power varied from 600, 900, 1200, and 1500 W. In addition, another experimental setup without laser heating was also conducted for comparison. Six forming passes with an angle increment of 15° were adapted to ensure the elimination of edge waves since it would interfere with evaluating the springback angle. The forming forces were also captured at a frame rate of 50 Hz during LRRF by a load sensor (OMEGA191, ATI Industrial Automation, Apex, USA). The forming parameters are summarized in Table 1.

### 2.2. Thermo-Mechanical Finite Element Model

**Figure 3:**
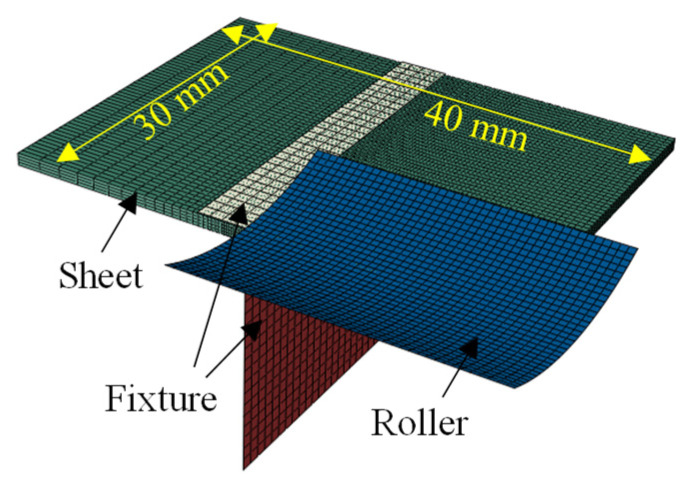
Geometry and mesh of the thermo-mechanical model.

To better understand the mechanical behavior during LRRF, a thermo-mechanical model was established using the commercial software, ABAQUS 2021. The geometric dimension of the finite element (FE) model is simplified for the sake of synthesized computational accuracy and efficiency, as presented in Figure 3. The fixtures and roller were defined as rigid bodies and meshed with 1-mm and 0.5-mm rectangular shell elements, respectively. The metal sheet was meshed with hexahedral solid elements. Four elements were adopted at the thickness direction and mesh size along the moving direction of the roller was 0.5 mm. Various mesh sizes were assigned along the transversal direction; the mesh sizes at the bending corner, which suffered from the most significant laser power density and most severe plastic deformation, were 0.2 mm, while the mesh sizes of the other area varied from 0.5 mm to 2 mm. Face-to-face contact between the metal sheet and the fixture and roller was used, and the default hard-contact modes in ABAQUS was adopted.

**Figure 4:**
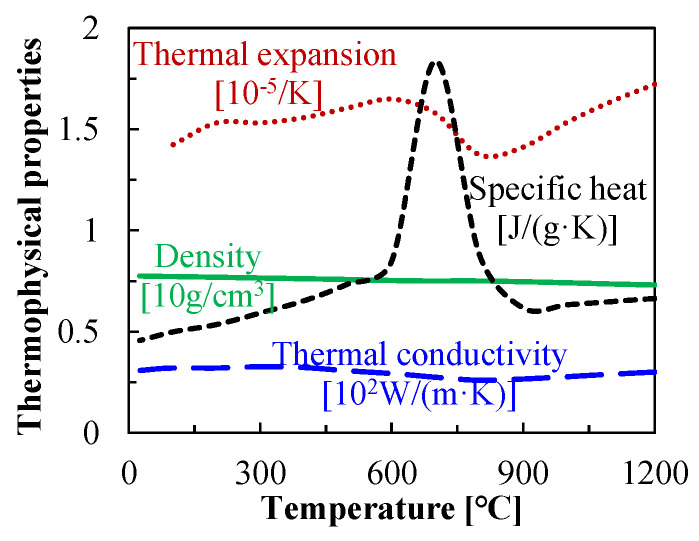
Thermophysical properties of QP1180 calculated using JMatPro.

The thermophysical properties of QP1180 steel were calculated using the JMatPro 7.0 software, as shown in Figure 4, according to the chemical compositions from Wang et al. [21].

**Figure 5:**
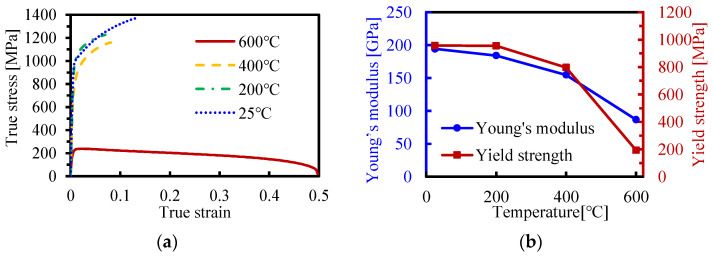
Temperature-dependent mechanical properties of QP1180 steel: (**a**) high-temperature flow-curves, and (**b**) temperature-dependent Young’s modulus and yield strength.

The thermo-mechanical properties of QP1180 steels were obtained by high-temperature quasi-static tensile tests at an MTS universal testing machine and the strains during tensile tests were captured with the aid of a digital image correlation (DIC) technique. Figure 5 demonstrates the flow curves and the mechanical properties of QP1180 at the temperatures of 25, 200, 400, and 600 °C. It was found that Young’s modulus and yield strength of QP1180 decrease with an increase in temperature, and a significant decrease is observed at the temperature range of 400–600 °C. The yield strength of QP1180 at 200 °C is almost identical to that at room temperature, indicating the dominant deformation mechanism of slip activity at this temperature range [22]. It is also interesting to note that the elongation and ultimate tensile strength of QP1180 decreases at 200 and 400 °C compared to that at room temperature; this is due to the fact that the transition from retained austenite to martensite is restricted at elevated temperatures, while the transformation induced plasticity (TRIP) effect is more prone to occur at room temperature [23]. QP1180 steels exhibit favorable elongation at 600 °C, which may be related to dynamic recovery and recrystallization at this temperature; in addition, an obvious softening effect was also notable at 600 °C, while a hardening effect still dominates below 400 °C.

Heat source models that define the laser power-density distribution are normally used to represent laser heating. There are diverse laser heat-source models relating to laser spot and process parameters. In this study, a combined Gaussian–uniform surface heat-source model was adopted to describe the rectangular laser spot, the power density (*q*) of which can be described as follows:

(1) $$q\left(x,y\right)=\frac{6\eta Q}{\sqrt{2\pi }ab}\cdot exp\left(-\frac{18x^{2}}{a^{2}}\right)\;\;\;\;\;\;\left(-\frac{b}{2}\leq y\leq \frac{b}{2}\right)$$

where *η* is laser absorption rate, *Q* is laser power, and *a*, *b* denotes the laser spot size. The heat source model was implemented into ABAQUS by user subroutine DFLUX. Thermal boundary conditions incorporating the thermal convection and radiation were considered. Details about the formulation of the heat source model and its validation through experiments can be found in [24] and are thus not repeated here.

## 3. Results

### 3.1. Experimental Results

**Figure 6:**
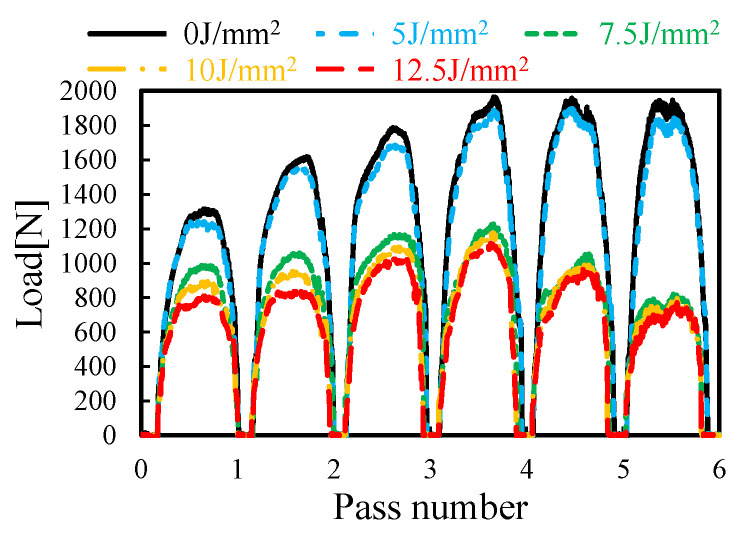
Effect of laser power density on the bending forces of LRRF.

The forming forces at each forming pass during LRRF are presented in Figure 6. The results indicate that there is no significant difference between forming with a laser power density of 5 J/mm^2^ in comparison with forming without laser heating. However, a remarkable decrease of ~43% in forming forces is notable when forming at a laser power density of more than 7.5 J/mm^2^. Besides, increasing laser power density above 7.5 J/mm^2^ has a negligible influence on the peak forming forces. The decrease in forming forces also reduces the demand on the load capacity and stiffness of the industrial robot and thus saves investment costs on the facilities. In addition, the laser power-density threshold between 5 J/mm^2^ and 7.5 J/mm^2^ also provides inspiration for the process optimization with limited laser energy. It was also found that the peak forming forces increase at the first four forming passes and then maintain or decrease levels for the last two forming passes. The increase in forming forces should be attributed to a larger plastic deformation with the cumulative forming passes in comparison to the participation of elastic deformation at the initial deformation stages, and the adequate softening effect at the last two forming passes might account for the constant or descending peak forming forces.

**Figure 7:**
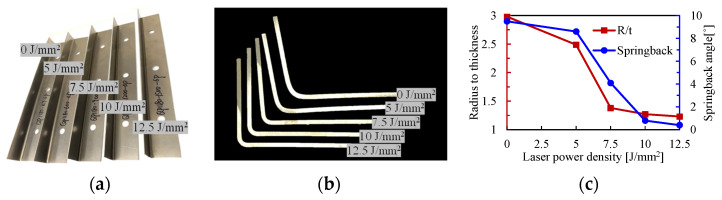
Effect of laser power density on the bending profiles of LRRF, (**a**) formed specimens, (**b**) cross-sectional profiles, (**c**) springback angle, and bending radius.

The formed specimens, the cross-sectional profiles of the formed L-shaped thin-walled parts and the corresponding *R/t* ratios and springback angles are presented in Figure 7. The decreasing *R/t* ratio and springback angle with the increase of laser power density are highly consistent with the decreasing forming forces; refer to Figure 6. The most significant improvements in bending radius and springback also happen at a laser power density between 5 and 7.5 J/mm^2^. There are also some minor differences, for example, the increase of laser power density from 0 to 5 J/mm^2^ can reduce the bending radius to some extent but not much on springback; the increasing laser power density between 7.5 and 10 J/mm^2^ improves the springback significantly while little improvement is seen for the bending radius. The bending radius as well as the springback angle could potentially be reduced with higher laser power density, while the increasing laser power density would bring about some other unwanted issues. For instance, the higher laser power density inevitably increases the peak temperature on the metal sheet, which would melt the metal sheet, and the subsequent solidification might lead to poor surface quality. Considering the requirement on compact profiles with small springback angle and the laser energy consumption, a laser power density of 10 J/mm^2^ was recommended, by which a small *R/t* ratio of ~1.2 and a negligible springback angle of 0.8° can be obtained.

### 3.2. Finite Element Simulation Results

**Figure 8:**
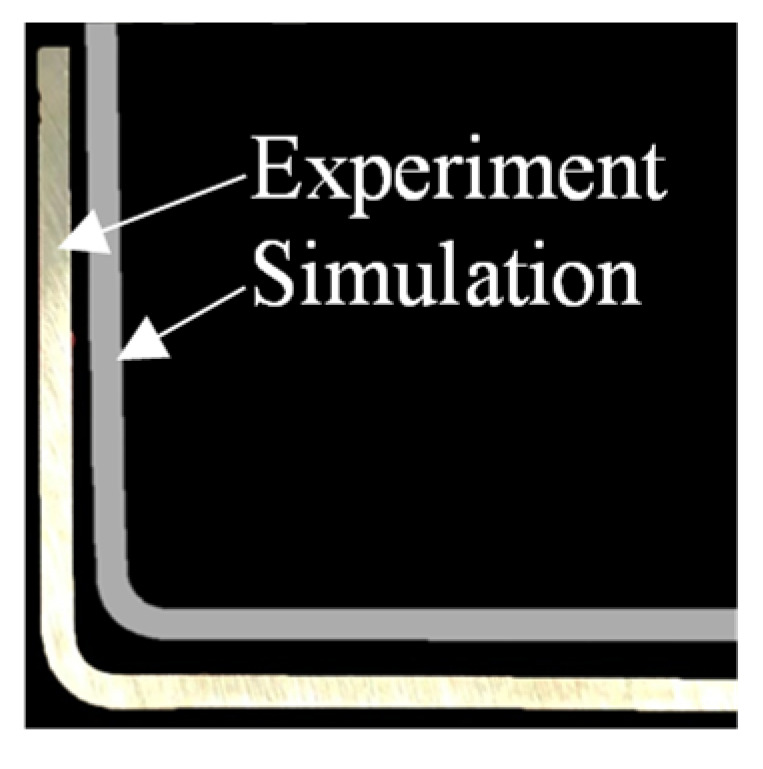
Experimental and predicted bending profiles of thin-walled parts formed at a laser power density of 10 J/mm^2^.

The predicted bending profile with a laser power density of 10 J/mm^2^ is given in Figure 8 together with the experimental profile. The predicted bending profile agreed with the experiments, and the deviations of the *R/t* ratio and springback angle was about ~15% and 0.2°, respectively.

**Figure 9:**
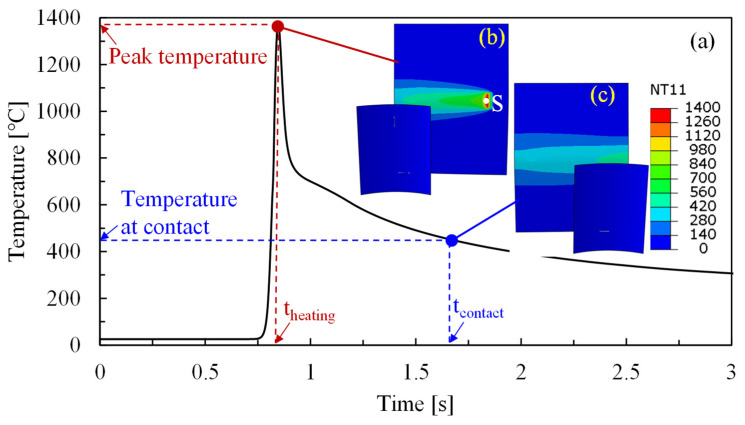
Temperature history and temperature field during LRRF at a laser power density of 10 J/mm^2^, (**a**) temperature history of one sampling point (point S in (**b**)) at the irradiated surface of the metal sheet; (**b**) temperature field at *t_heating_*; (**c**) temperature field at *t_contact_*, where *t_heating_* is the time when laser is imposed directly on the sampling point and *t_contact_* is the time when the roller is in contact with the same position.

The temperature field and temperature history at the laser-irradiated surface of the metal sheet formed at a laser power density of 10 J/mm^2^ is shown in Figure 9. It is worth mentioning that there is always a time difference between laser heating and forming due to the distance between the laser spot and roller, and the forming time is always delayed compared with the heating time. Therefore, two images are presented in Figure 9 to detail the difference. Figure 9b demonstrates the relative position between the laser spot and the roller. It can be found that the roller was not directly in contact with the position where the laser imposes or the position with peak temperature. It can also be observed in Figure 9c that when roller moves to the above-mentioned position, the temperature has already decreased significantly. Two periods were introduced to quantify the changes in temperature: the time when one position at the metal sheet reaches its peak temperature is termed as heating time (*t_heating_*), and the time when the same position makes direct contact with the roller is named the contact time (*t_contact_*). As a result, Figure 9b,c represent the temperature field at *t_heating_* and *t_contact_*, respectively. More specifically, the time interval between the heating time and contact time can be calculated as follows:

(2) $$t_{contact}-t_{heating}=d/v$$

where *d* is the distance between laser spot and roller, and *v* indicates laser scanning speed, which is also the moving speed of the roller. Consequently, the temperature history at laser spot center in Figure 9b was extracted from the FE model and then presented in Figure 9a. The temperature vs. time curve suggests a higher cooling rate at higher temperatures, and the temperature at *t_contact_* is about 450 °C, although the peak temperature reaches ~1370 °C at *t_heating_*. The temperature value at *t_contact_* also implies the minimum forming temperature since the largest bending angle is achieved at *t_contact_* by the direct contact between the roller and this position. Therefore, forming can be regarded as taking place at the time intervals between *t_heating_* and *t_contact_* or within the temperature range between peak temperature and temperature at contact in Figure 9a. It is, of course, possible to tune the time intervals or temperature ranges by altering the distance between the laser spot and roller or the scanning speed, as suggested in Equation (2); however, this is beyond the scope of this study.

## 4. Discussion

### 4.1. Decrease of Bending Forces

**Figure 10:**
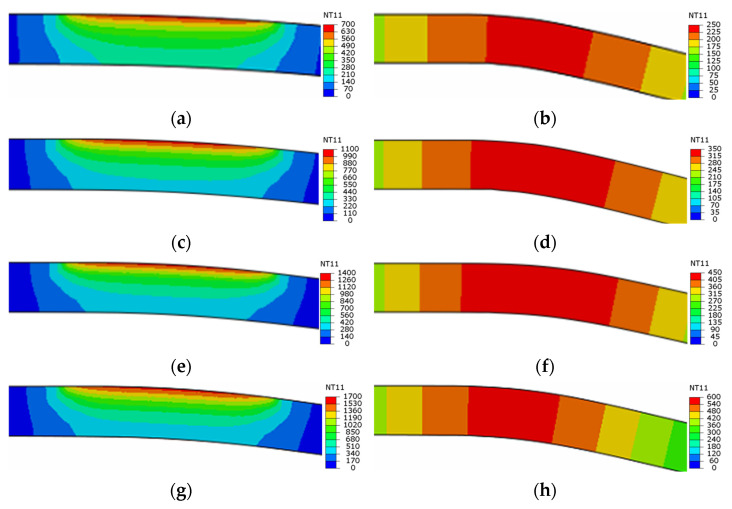
Cross-sectional temperature field of metal sheets formed at laser power densities of 5 J/mm^2^ (**a**,**b**), 7.5 J/mm^2^ (**c**,**d**), 10 J/mm^2^ (**e**,**f**), and 12.5 J/mm^2^ (**g**,**h**). Here (**a**,**c**,**e**,**g**) indicate temperature fields at *t_heating_*, and (**b**,**d**,**f**,**h**) denote temperature fields at *t_contact_*.

As declared in Section 3.2, forming mainly takes place between *t_heating_* and *t_contact_*; therefore, the temperature field at this time interval is quite important for understanding the effect of laser power density on bending forces during LRRF. Since the laser is a one-of-a-kind centralized heat source, the temperature distribution is quite uneven in the metal sheet; the cross-sectional temperature fields at *t_heating_* and *t_contact_* with different laser power densities are thus given in Figure 10. Note that only the temperature field at the first forming pass is presented here as an example. The obvious temperature gradients from both the transversal direction and thickness direction at *t_heating_* can be noticed at any laser power density, however, the temperature distribution is relatively uniform at least from the thickness direction at *t_contact_* thanks to the thermal conduction. It is also found that the deformation temperatures vary from 250–680 °C, 350–1040 °C, 450–1370 °C, and 560–1630 °C when forming at laser power densities of 5 J/mm^2^, 7.5 J/mm^2^, 10 J/mm^2^, and 12.5 J/mm^2^, respectively. It has been proved in Figure 5 that the yield strength of QP1180 steel significantly decreases at temperatures above 400 °C, which is likely responsible for the obvious decrease in bending force at a laser power density of more than 7.5 J/mm^2^. In addition, partial austenitization occurs on the metal sheet at temperatures above 874 °C, according to JMatPro. Austenite should then transform into fresh martensite again due to the subsequent fast cooling [4], and the critical martensitic transformation temperature (M_s_) was estimated to be 339 °C according to JMatPro. It is interesting to find that the peak temperatures exceeded the austenitization temperature while forming temperatures were always above the M_s_ temperature when forming at a laser power density of more than 7.5 J/mm^2^, suggesting the existence of austenite during forming, and this is likely also one of the major factors that decrease the bending forces. While, for another case of forming at a laser power density of 5 J/mm^2^, the peak temperature was below the austenitization temperature, a forming temperature between 250–680 °C may provide limited help in reducing the bending forces. 

### 4.2. Reduction in Springback

**Figure 11:**
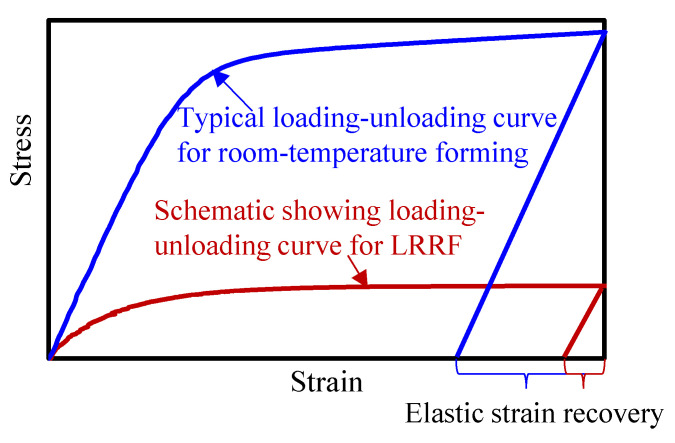
Schematic showing different loading-unloading curves for room-temperature forming and LRRF. Room-temperature forming: loading with larger stresses and unloading with the same Young’s modulus; LRRF: loading with smaller stresses and relatively larger Young’s modulus at unloading than at loading.

Springback normally happens at the end of the forming process due to the inevitable elastic-strain recovery after the release of the forces of forming tools, as schematically shown in Figure 11. There are two crucial factors that affect the springback angle: the stress at loading and Young’s modulus after unloading. Springback is typically more severe with larger stresses and a smaller Young’s modulus. In room-temperature forming, the ultrahigh-strength metal sheet was deformed at large stresses and the loading and unloading Young’s modulus were generally constant. In our LRRF cases, real-time unloading occured with the motion of the forming roller, and the time when the roller is in contact with the blank, i.e., above-mentioned *t_contact_*, could be regarded as the threshold of loading and unloading. Consequently, the stress of material at *t_contact_* and the Young’s modulus after *t_contact_* have a direct impact on the springback angle, which in turn, highly rely on the temperature at and after *t_contact_*. It can be found in Figure 10 that the temperatures at *t_contact_* or at the beginning of unloading are 250, 350, 450, and 560 °C, respectively, when forming with laser power densities of 5, 7.5, 10, and 12.5 J/mm^2^. The yield strength of QP1180 at about 200 °C was almost the same as that at room temperature, thus, the springback angle rarely changes with a laser power density of 5 J/mm^2^. The visible decrease in springback angle at 10 J/mm^2^ is also consistent with the lower yield strength at the temperature range of 400–600 °C. Although both the yield strength and Young’s modulus are decreased at elevated temperature, the loading and unloading always happened gradually with the motion of the roller instead of instantaneously. Loading occured at a time interval between *t_heating_* and *t_contact_*, while unloading was accompanied by cooling or the recovery of Young’s modulus after *t_contact_*, leading to a larger Young’s modulus at unloading than that at loading, as shown in Figure 11; thus, the increase in springback angle due to the decrease of Young’s modulus compared to that at room temperature was not observed. It is important to emphasize that Figure 11 illustratively presents the different Young’s modulus values at loading and unloading of LRRF, and the actual loading-unloading or stress vs. strain curve is much more complicated since LRRF is a non-isothermal forming process and Young’s modulus always varies over time. The mechanism of loading at higher temperatures and unloading at lower temperatures is exactly one of the advantages of the LRRF process. However, it is still worth mentioning that the influence of decreasing Young’s modulus cannot be completely ignored. As can be seen in Figure 7b, the springback angles between metal sheets formed at laser power densities of 10 and 12.5 J/mm^2^ are quite similar, although the temperatures at unloading vary from 450 and 560 °C and the yield strength is undoubtfully different. The slight difference in springback angle in spite of significant temperature difference implies that there should be a balance between the yield strength and Young’s modulus.

### 4.3. Formation Mechanism of Sharp Bending Radius

**Figure 12:**
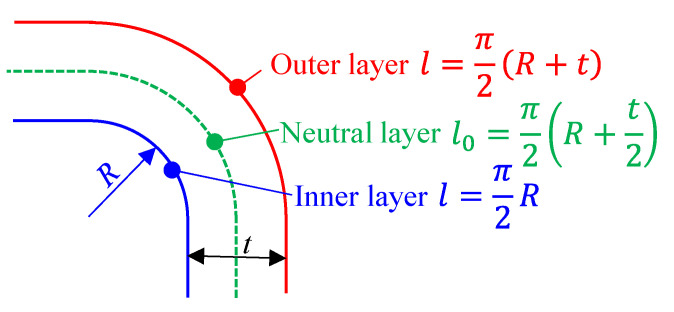
Schematic of an ideal 90-degree bending angle.

For an ideal 90-degree bending angle, the total plastic strains at the outer and inner layer of the bending corner can be calculated according to Equation (3).

(3) $$\varepsilon =\frac{\left|l-l_{0}\right|}{l_{0}}=\frac{1}{2\times \frac{R}{t}+1}$$

where *l* is the length of the outer and inner layer after deformation and *l*_0_ is the initial length of outer and inner layer, or the length of a neutral layer (not changed ideally), as shown in Figure 12. Although the actual bending process can slightly differ in thickness variation or neutral layer shifting, this equation still suggests that larger strains are always required to form thin-walled parts with smaller *R/t* ratios, which is more likely to happen in LRRF due to the softening effect of laser heating.

**Figure 13:**
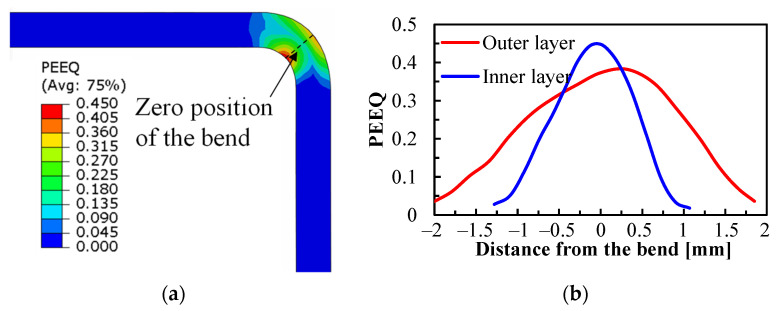
PEEQ of metal sheet formed at a laser power density of 10 J/mm^2^, (**a**) cross-sectional PEEQ distribution, (**b**) PEEQ value extracted from the outer and inner layer of the bend.

The equivalent plastic strain (PEEQ) after forming at a laser power density of 10 J/mm^2^ is presented in Figure 13a. The outer and inner layers at the corner suffer from the most significant tensile and compression stresses, respectively, and thus the maximum PEEQ always appears at these two locations. The PEEQ distributions along the transversal direction are extracted from both the outer and inner layers, as shown in Figure 13b. It can be found that the PEEQ distribution is quite concentrated at the bending corner. The introduction of laser heating actually induces large temperature gradients, especially through the transversal direction, as shown in Figure 10, and consequently results in a gradient of mechanical properties at the bending corner. The area that suffers from the maximum laser energy input has much lower strength, as well as lower deformation resistance, and the plastic deformation is thus inclined to take place at the heating area. In terms of forming at room temperature, the mechanical properties are homogeneous and, therefore, a larger area tends to be deformed at the same time as the initial deformation stage. In addition, the plastic deformation at room temperature also leads to work hardening and, thus, a larger deformation resistance at the already-deformed area, and localized deformation is not likely to happen.

## 5. Conclusions

In this research, a laser-assisted robotic roller forming was applied to bend ultrahigh-strength steels to thin-walled parts with a smaller bending force, springback angle, and bending radius. The effect of laser power density on the bending process and final part profiles were experimentally investigated and also explained using thermo-mechanical modeling. The main conclusions are summarized below:

(1) Bending forces, springback angle, and bending radius in LRRF can be reduced simultaneously with higher laser power density, whereas an optimized parameter of forming at a laser power density of 10 J/mm^2^ is recommended since the improvement is limited with the continuous increase of laser energy.
(2) The decrease in bending force can be attributed to a lower yield strength. The occurrence of austenitization and subsequent deformation before martensitic transformation also play a significant part in reducing bending forces.
(3) The mechanism of forming at higher temperatures and unloading at lower temperatures is beneficial for reducing springback.
(4) Localized deformation is more likely to happen with partial laser heating thanks to the mechanical properties induced by temperature gradients, promoting the formation of small bending radius.

This research focuses on the effect of laser power density on bending forces and part profiles based on the fact that laser power density has the most significant influence on the temperature field in laser-assisted forming. Optimization of other process parameters, e.g., forming pass numbers and angle increments at each forming pass, would also be very promising for obtaining the ideal component parameters satisfying the requirement of safety and compact design, and this will be the objective of further investigation.

## Figures and Tables

**Table 1:** Laser-assisted robotic roller forming parameters.

Laser Power Density	Laser Power	Laser Scanning Speed	Laser Spot Size	Forming Angle
0 J/mm^2^	N/A	30 mm/s	4 mm × 2 mm	15° × 6 passes
5 J/mm^2^	600 W			
7.5 J/mm^2^	900 W			
10 J/mm^2^	1200 W			
12.5 J/mm^2^	1500 W			

## Data Availability

Not applicable.
